# Cruciferous vegetables (*Brassica oleracea*) confer cytoprotective effects in *Drosophila* intestines

**DOI:** 10.1080/19490976.2021.1921926

**Published:** 2021-05-08

**Authors:** James T. Lyles, Liping Luo, Ken Liu, Dean P. Jones, Rheinallt M. Jones, Cassandra L. Quave

**Affiliations:** a Center for the Study of Human Health, Emory University College of Arts and Sciences, Atlanta, Georgia; b Department of Pediatrics, Emory University School of Medicine, Atlanta, Georgia; c Division of Pulmonary, Allergy, Critical Care and Sleep Medicine, Emory University School of Medicine, Atlanta, Georgia; d Department of Dermatology, Emory University School of Medicine, Atlanta, Georgia

**Keywords:** Indole, *Brassica*, *Drosophila*, nrf2, leaky-gut

## Abstract

Varieties and cultivars of the cruciferous vegetable *Brassica oleracea* are widely presumed to elicit positive influences on mammalian health and disease, particularly related to their indole and sulforaphane content. However, there is a considerable gap in knowledge regarding the mechanisms whereby these plant-derived molecules elicit their beneficial effects on the host. In this study, we examined the chemical variation between *B. oleracea* varieties and evaluated their capacity to both activate Nrf2 in the *Drosophila* intestine and elicit cytoprotection. Ten types of edible *B. oleracea* were purchased and *B. macrocarpa* was wild collected. Fresh material was dried, extracted by double maceration and green kale was also subjected to anaerobic fermentation before processing. Untargeted metabolomics was used to perform Principal Component Analysis. Targeted mass spectral analysis determined the presence of six indole species and quantified indole. Extracts were tested for their capacity to activate Nrf2 in the *Drosophila* intestine in third instar *Drosophila* larvae. Cytoprotective effects were evaluated using a paraquat-induced oxidative stress gut injury model. A “Smurf” assay was used to determine protective capacity against a chemically induced leaky gut. Extracts of Brussels sprouts and broccoli activated Nrf2 and protected against paraquat-induced damage and leaky gut. Lacto-fermented kale showed a cytoprotective effect, increasing survival by 20% over the non-fermented extract, but did not protect against leaky gut. The protective effects observed do not directly correlate with indole content, suggesting involvement of multiple compounds and a synergistic mechanism.

## Introduction

*Brassica oleracea* L. (Brassicaceae) is a cruciferous vegetable used worldwide as a food and traditional medicine. There are many varieties, and these vegetables are among the oldest cultivated plants known. Indoles, which are enriched in *Brassica* spp., extend the healthspan of diverse phyla by modulating sensitivity to stressors, extending reproductive span, and increasing motility in the aged.^1^

The nuclear factor erythroid-related factor 2 (Nrf2) pathway is a conserved signaling pathway known for its role in orchestrating the primary antioxidant response in cells during electrophilic and oxidative stress. Within the context of the gastrointestinal tract, altered Nrf2-signaling is at the forefront of acute and chronic colitis, including ulcerative colitis and Crohn’s disease, drug-related injuries, and hepatobiliary and gastrointestinal carcinogenesis.

Conceptualized as a pathway for the host response to reactive oxygen species and electrophiles, the Nrf2/ARE signaling module emerged as a central cellular signal transduction pathway that is also responsive to xenobiotics.^2^ Nrf2 is a DNA binding transcription factor that is constitutively ubiquitinated and targeted for degradation by the activity of the E3 ligase KEAP-1. Oxidant and electrophilic stress is perceived by KEAP-1 via thiol reactive cysteines, which, when oxidized or alkylated, induces a conformational change in KEAP-1 that results in the release of Nrf2, cytoplasmic accumulation, and ultimate translocation to the nucleus. Because the Nrf2 pathway is fully developed in *Drosophila melanogaster*, it is a faithful model organism to study how indole-containing *B. oleracea* varieties ensure mucosal barrier integrity and mitigate damage severity in response to pathogens, environmental stressors, or immune responses and on functional outputs at the organismal level.

## Results

In order to understand the cytoprotective effects of cruciferous vegetables, different *B. oleracea* varieties were extracted and tested for Nrf2 activation in *Drosophila*, cytoprotective capacity in a gut injury model, and their ability to inhibit leaky gut. The extracts were evaluated by mass spectrometry for the presence of indole compounds.

### *Metabolomics of Brassica* spp. *extracts*

The amount of indole present in the *Brassica* spp. extracts was quantified and other detected indole species are indicated in Table 1. For the *Brassica oleracea* varieties, Kohlrabi, fermented kale, fresh kale, and broccoli had the highest concentration of indole with 2.32, 2.14, 1.67, and 1.64 μg indole/g dry plant material. All *B. oleracea* varieties contained indole, IAA, I3CA, ICA, and I3C. DIM was only found in Brussels sprouts, fresh and fermented green kale.

**Table 1.** Quantitation of indole and identification of other indoles by targeted UPLC-HRMS. The concentration of indole in *Brassica* spp. is reported as mg indole/g of dry plant material and the other indoles detected in the *Brassica* spp. are indicated by **+**: detected in the extract or -: not detected

Common Name (Scientific Name)	Indole	IAA	I3CA	DIM	ICA	I3C
Wild Mediterranean species (*Brassica macrocarpa* Guss.)	+^a^	+	+	-	+	+
Broccolini (*B. oleracea* Italica Group x Alboglabra Group)	1.21	+	+	-	+	+
Broccoli (*B. oleracea* var. *italica*)	1.64	+	+	-	+	+
Brussels Sprouts (*B. oleracea* var. *gemmifera*)	1.29	+	+	+	+	+
Green Cabbage (*B. oleracea* var. *capitata*)	1.16	+	+	-	+	+
Red Cabbage (*B. oleracea* var. *capitata* f. *rubra*)	0.81	+	+	-	+	+
Savoy Cabbage (*B. oleracea* var. *capitata* f. *sabauda*)	0.97	+	+	-	+	+
Cauliflower (*B. oleracea* var. *botrytis*)	1.21	+	+	-	+	+
Collard Greens (*B. oleracea* var. *viridis*)	0.90	+	+	-	+	+
Dinosaur Kale (*B. oleracea* var. *palmifolia*)	1.37	+	+	-	+	+
Green Kale, fermented (*B. oleracea* Acephala Group)	2.14	+	+	+	+	+
Green Kale, fresh (*B. oleracea* Acephala Group)	1.67	+	+	+	+	+
Red Kale (*B. oleracea* Acephala Group)	0.50	+	+	-	+	+
Kohlrabi (*B. oleracea* var. *gongylodes*)	2.32	+	+	-	+	+

IAA: indole-3-acetic acid; I3CA: indole-3-carboxylic acid; DIM: 3,3-diindolylmethane, ICA: indole-3-carboxaldehyde; I3C: indole-3-carbinol.

^a^Indole detected by UPLC-HRMS, but not quantified in sample.

**Figure 1.**
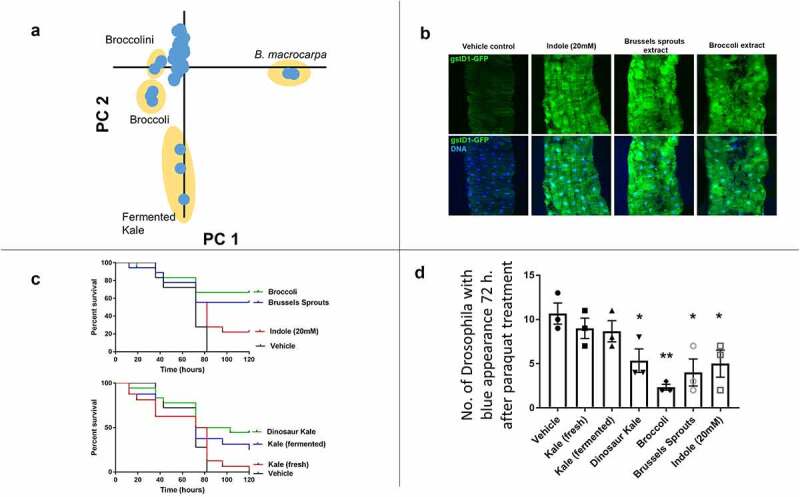
(a) PCA Scores plot generated from the negative ESI-C18 UPLC-MS data showing lacto-fermented kale, broccolini, broccoli, and *B. macrocarpa* as chemically distinct from other *Brassica* spp. All extracts were run in triplicate. (b) Indole or *B. oleracea* extracts induce Nrf2 signaling in the Drosophila intestine. Detection of GFP under the transcriptional control of a Nrf2-responsive element in third instar *Drosophila* larvae fed either indole (20 mM), or 10 mg/mL extracts of Brussels sprouts or broccoli extract for 4 hours. Note activation of Nrf2 responsive GFP following ingestion or indole, Brussels sprouts, or broccoli. (c) The *B. oleracea* varieties extracts confer potent cytoprotective effects in Drosophila intestine in response to an oxidative challenge. Graphs show the relative survival of Drosophila fed the indicated *B. oleracea* varieties extracts in response to paraquat challenge. Note the significantly enhanced survival of flies fed extracts of broccoli or Brussels sprouts and dinosaur kale or lacto-fermented kale. The Log rank (Mantel-Cox) test for vehicle vs. broccoli p ≤ 0.0001, n = 20, vehicle vs. Brussels sprouts p ≤ 0.0004, n = 20, vehicle vs. indole p ≤ 0.0527, n = 20, vehicle vs. dinosaur kale p ≤ 0.0262, n = 20, and vehicle vs. kale p ≤ 0.3819, n = 20. (d) Extracts of *B. oleracea* varieties protect against a leaky-gut phenotype. The *Drosophila* were fed *B. oleracea* extracts for 3 days, then were subjected to a paraquat solution, spiked with blue dye, which induces a leaky gut phenotype. Flies that have a leaky gut at 72 hours are identified by exhibiting a visible blue color throughout their hemocoel within the body cavity. Nonparametric unpaired t-test *p < .05, **p < .01

In the PCA analysis, most *Brassica* extracts group together (Figure 1a). *B. macrocarpa* is a wild, non-domesticated relative of *B. oleracea*. Since domesticated plant species are less chemically diverse than their wild relatives,^3^ *B. macrocarpa* serves as an outlier in the PCA analysis. The leaky gut-protecting extracts from fermented kale, broccoli, and broccolini appear separated in the scores plots from *B. macrocarpa* and the other domesticated species. The fermented kale appears distinct from all the other extracts, which included the non-fermented kale. Not only is the overall chemistry of this fermented product different from the fresh sample but it is also chemically distinct from all other *Brassica* extracts.

### Cytoprotection against paraquat-induced oxidative stress *Drosophila* gut injury model

Broccoli and Brussels sprouts extracts were potent activators of the Nrf2/CncC pathway compared to control *Drosophila* fed purified indole, which has documented inducement of Nrf2 signaling (Figure 1b).^4^ *B. oleracea* extracts also elicited cytoprotective influences against paraquat-induced oxidative challenge. Flies that ingested broccoli or Brussels sprouts extracts were protected against oxidative injury as determined by increased survival in response to paraquat (Figure 1c). Other extracts did not elicit cytoprotection to the same extent, indicating the beneficial effect is variable among *B. oleracea* varieties. The capacity of *B. oleracea* extracts to modulate gut permeability in adult *Drosophila* was assessed in corroborating survival assays (Figure 1c). Supplementation with broccoli or Brussels sprouts extracts resulted in significantly fewer flies with blue dye translocation to the hemolymph (Figure 1d). These data demonstrate the first evidence for the beneficial properties of broccoli or Brussels sprouts extracts, which were markedly more efficacious compared to the other varieties tested.

## Discussion

The paraquat-induced oxidative stress gut injury in *Drosophila* model screens for beneficial agents that elicit gut cytoprotection.^5^ Only Brussels sprouts and broccoli activated Nrf2 and protected against paraquat-induced damage and leaky gut. DIM was only identified in Brussels sprouts, fermented and fresh kale. Of these, the broccoli provided more cytoprotection in the gut and was more protective against leaky gut syndrome (Figure 1c,d). Broccoli was the most chemically distinct from all the other unfermented *B. oleracea* extracts (Figure 1a). Additional research into the chemical differences of this variety and their effect on the biological activity is warranted.

Lacto-fermented kale showed a cytoprotective effect, increasing survival by 20% over the non-fermented extract (Figure 1c), but neither kale extract protected against leaky gut. The lacto-fermented kale is distinct from other *B. oleracea* species in the PCA analysis, including the unfermented kale (Figure 1a). Therefore, the process of lacto-fermentation either yields new biologically active compounds, degrades compounds which may interfere with the tested bioactivity, or increases the bioavailability of the active compounds. Future work should be directed to understanding the nature of this cytoprotection, how it was enhanced by fermentation, and to what extent DIM may play a role. Lacto-fermented *B. oleracea* foods are generally associated with concepts of “health promotion.”^1^ The increases in *Drosophila* survival for fermented kale supports this concept, at least in lower organisms.

Indole alone elicited a positive effect in all three models in line with other recent studies of indoles in models of healthspan^1^ and aging;^6^ however, in our study, the indole response was never as strong as the effect observed in the chemically complex plant extracts. Since 10 of the 12 non-fermented domestic *Brassica* clustered together in the PCA analysis and the targeted analysis showed little variation in the presence of six indoles investigated, the observed variations in cytoprotection are likely due to other molecules. As the protective effects of the tested extracts do not directly correlate with indole presence, the benefit conferred by these vegetables is more complex, likely involving several compounds and a synergistic mechanism.

## Materials and methods

### Generation of *B. oleracea* extracts

**Figure 2.**
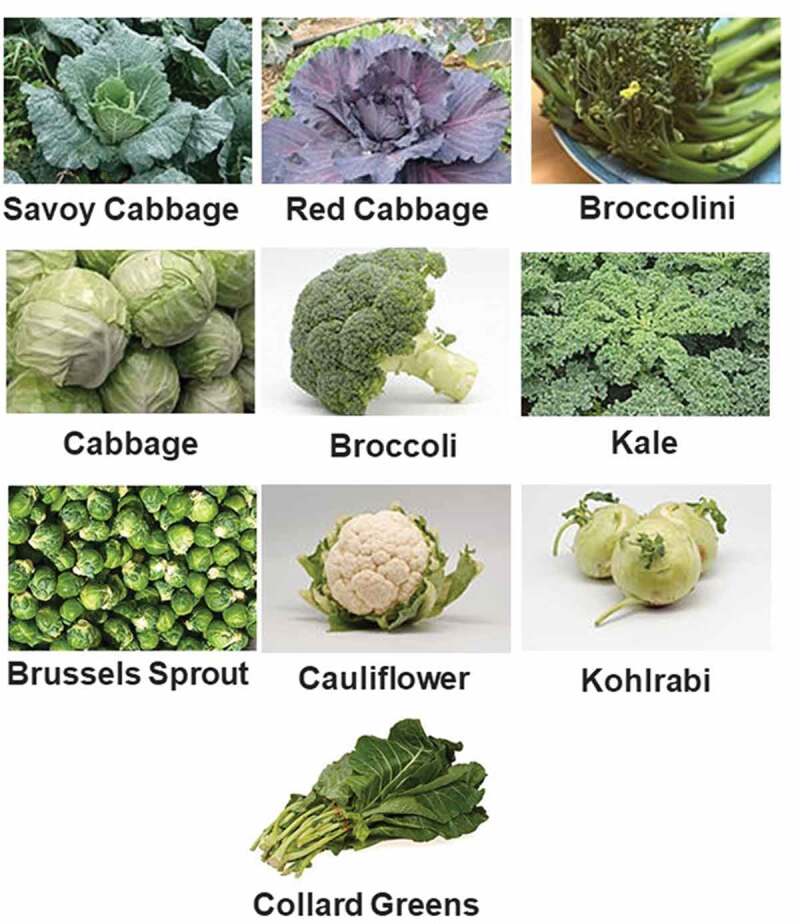
Examples of the diversity of edible *Brassica oleracea* varieties

Ten fresh, organic varieties of *Brassica oleracea* (Figure 2) were purchased from The Dekalb Farmers Market (Atlanta, GA). The wild collected Mediterranean species, *Brassica macrocarpa*, was collected in accordance with WHO GACP guidelines^7^ and botanical vouchers deposited at the Emory University Herbarium (GEO-MB-022133-0 and GEO-MB-022134-0). The fresh material was diced and dried in a desiccating cabinet, 35–40°C. The dried material ground through a 2 mm mesh using a Thomas Scientific Wiley Mill. The resulting powder extracted by double maceration for 72 hours in 80% ethanol_(aq)_ at a ratio of 1 g dry powder:10 mL solvent. Each extract’s macerations were combined, concentrated in vacuo at <40°C, and lyophilized. The resulting extract was stored at −20°C until analysis. Additionally, fresh kale was subjected to anaerobic lactic acid bacteria fermentation using 3% brine solution for 7 days at room temperature in a pickling vessel equipped with a one-way air lock. Following the fermentation period, the plant material was removed from the brine, lyophilized, and processed as described above.

### High-resolution metabolomics assessment and Quantitation of *Brassica* spp. extracts

Metabolomic assessment of the *Brassica* spp. extracts was performed on a Dionex Ultimate 3000 UPLC coupled to Thermo Scientific Orbitrap Fusion Tribrid mass spectrometer. The samples were analyzed using HILIC with positive mode ESI and reverse phase C18 chromatography with negative mode ESI. Data dependent MS/MS (ddMSMS) was collected for the top 10 features detected per scan.

The presence of six indole species, indole, indole-3-acetic acid (IAA), indole-3-carboxylic acid (I3CA), 3,3′-diindolylmethane (DIM), indole-3-carboxaldehyde (ICA), and indole-3-carbinol (I3C), was qualitatively determined using targeted MS/MS for the indoles. Putative matches were compared with retention time and MS^2^ data for authentic standards.

Quantification of indole was performed using the previously described LC-MS conditions and quantified using the method of reference standardization as described by Lui et al.^8^

### Drosophila paraquat resistance assays

The model organism *Drosophila* was used to study cytoprotection in response to methyl viologen dichloride (paraquat)-induced oxidative stress.^9,10^ Groups of 20, 5-day-old adult *Drosophila* of assayed genotypes were fed a solution containing a semi-lethal dose of paraquat (17.5 mM). Percent of surviving flies were recorded and compared by log-rank Martel–Cox test using Graphpad Prism.

### Assessment of gut permeability in adult Drosophila assay

Since indole-fed *Drosophila* have established Nrf2 signaling, the gut permeability in adult *Drosophila* was undertaken based on assays described in Darby et al.^5^ Briefly, male *w1118* flies were aged to 5-days old. The fly food was supplemented with *Brassica* extracts for 3 days. Then, flies were subjected to 17.5 mM paraquat solution spiked with blue dye which induces a leaky gut phenotype. Flies that have a leaky gut are identified by a visible blue color within their hemocoel. Flies were anesthetized daily with CO_2_ and visually scored for blue dye infiltration and the ‘Smurf’-like phenotype.

## References

[1] Sonowal R, Swimm A, Sahoo A, Luo L, Matsunaga Y, Wu Z, Bhingarde JA, Ejzak EA, Ranawade A, Qadota H, et al. Indoles from commensal bacteria extend healthspan. PNAS. 2017;114:E7506–6. 28827345 10.1073/pnas.1706464114 PMC5594673

[2] Maher J, Yamamoto M. The rise of antioxidant signaling – the evolution and hormetic actions of Nrf2. Toxicol Appl Pharmacol. 2010;244:4–15. doi:10.1016/j.taap.2010.01.011. 20122947

[3] Moreira X, Abdala-Roberts L, Gols R, Francisco M. Plant domestication decreases both constitutive and induced chemical defences by direct selection against defensive traits. Sci Rep. 2018;8:12678. doi:10.1038/s41598-018-31041-0. 30140028 PMC6107632

[4] Sita G, Hrelia P, Graziosi A, Morroni F. Sulforaphane from cruciferous vegetables: recent advances to improve glioblastoma treatment. Nutrients. 2018;10(11):1755. doi:10.3390/nu10111755. PMC6267435 30441761

[5] Darby TM, Owens JA, Saeedi BJ, Luo L, Matthews JD, Robinson BS, Naudin CR, Jones RM. *Lactococcus lactis* subsp. *cremoris* is an efficacious beneficial bacterium that limits tissue injury in the intestine. iScience. 2019;12:356–367. doi:10.1016/j.isci.2019.01.030. 30739017 PMC6369221

[6] Powell DN, Swimm A, Sonowal R, Bretin A, Gewirtz AT, Jones RM, Kalman D. Indoles from the commensal microbiota act via the AHR and IL-10 to tune the cellular composition of the colonic epithelium during aging. Proc Natl Acad Sci. 2020;117:21519–21526. doi:10.1073/pnas.2003004117. 32817517 PMC7474656

[7] WHO. WHO guidelines on good agricultural and collection practices (GACP) for medicinal plants. Geneva; 2003.

[8] Liu KH, Nellis M, Uppal K, Ma C, Tran V, Liang Y, Walker DI, Jones DP. Reference standardization for quantification and harmonization of large-scale metabolomics. Anal Chem. 2020;92:8836–8844. doi:10.1021/acs.analchem.0c00338. 32490663 PMC7887762

[9] Jones RM, Desai C, Darby TM, Luo L, Wolfarth AA, Scharer CD, Ardita C, Reedy A, Keebaugh E, Neish A, et al. Lactobacilli modulate epithelial cytoprotection through the Nrf2 pathway. Cell Rep. 2015;12:1217–1225. doi:10.1016/j.celrep.2015.07.042. 26279578 PMC4640184

[10] Luo L, Reedy AR, Jones RM. Detecting reactive oxygen species generation and stem cell proliferation in the Drosophila intestine. In: Ivanov A, editor. Gastrointestinal physiology and diseases. New York (NY): Humana Press; 2016. p. 103–113. 10.1007/978-1-4939-3603-8_10 27246026

